# Effect of Starchy Wall Materials in the Microencapsulation of Carambola Fruit (*Averrhoa carambola*) Pulp: Antioxidant Characteristics

**DOI:** 10.3390/foods15101699

**Published:** 2026-05-12

**Authors:** Reyna S. Santana-Galeana, Jose Alvarez-Ramirez, Edith Agama-Acevedo, Apolonio Vargas-Torres, Luis A. Bello-Pérez

**Affiliations:** 1 Instituto Politécnico Nacional (IPN), Centro de Desarrollo de Productos Bióticos (CEPROBI), Yautepec 62731, Morelos, Mexico; rsantana2400@alumno.ipn.mx (R.S.S.-G.); eagama@ipn.mx (E.A.-A.); 2 Departamento de Ingeniería de Procesos e Hidráulica, Universidad Autónoma Metropolitana, Mexico City 09340, Iztapalapa, Mexico; jjar1963@gmail.com; 3 Instituto de Ciencias Agropecuarias, Centro de Investigación en Ciencia y Tecnología de Alimentos, Universidad Autónoma del Estado de Hidalgo, Tulancingo 43600, Hidalgo, Mexico; apolovt@hotmail.com

**Keywords:** *Averrhoa carambola*, polyphenols, antioxidants, microcapsules

## Abstract

*Averrhoa carambola*, known as Carambola or Star fruit, was collected in a semitropical region of central Mexico. Extractable and non-extractable polyphenols were characterized in terms of their antioxidant capacity (ABTS+, DPPH, and FRAP). Extractable polyphenols showed a higher antioxidant capacity than their non-extractable counterparts, with values of 65.61, 109.37, and 83.90 μmol TE/g db for ABTS+, DPPH, and FRAP, respectively. These values are consistent with reports on fruit cultivated in Asia. Maltodextrin-based microcapsules, N-Lok and Capsul, were prepared to protect the extract from adverse conditions. The ability of the microcapsules to protect polyphenols and their antioxidant capacity was quantified by half-life, which ranged from 10 to 50 days. Principal component analysis was performed to evaluate the multivariate relationships between polyphenol half-life and antioxidant capacity in the microcapsules. The results showed that no single microcapsule could provide the best protection across all polyphenol content and antioxidant capacity levels. The main conclusion is that the type of capsule material depends on the specific application, particularly on which antioxidant properties are desired.

## 1. Introduction

*Averrhoa carambola*, commonly known as Carambola or Star fruit, is a tropical evergreen tree whose fruit has a distinctive star shape in cross-section. This fruit is valued for its sweet and sour flavor and low calorie content [1]. Carambola is widely cultivated in tropical and subtropical regions worldwide. Malaysia is a world leader in both production volume and exports of high-quality Carambola to Europe and Asia [2]. Other major producers include Taiwan, Thailand, Indonesia, and Brazil. In the United States, it is commercially grown in parts of Florida and Hawaii.

Acidic varieties are typically smaller, more flavorful, and have a higher oxalic acid content. Sweet varieties, on the other hand, are larger, with a milder flavor and less acidity. The main problem with Carambola fruit is oxidation, which reduces its shelf life. The use of the pulp to produce a functional ingredient with antioxidant compounds highlights the circular economy concept. Star fruit is a low-calorie fruit, rich in water, dietary fiber, and vitamin C. Plant compounds present in the fruit have antioxidant properties and have been suggested to help control cholesterol. The beneficial effects of Carambola fruit include antioxidant (mediated via L-ascorbic acid, epicatechin, and Gallic Acid), hypoglycemic (mediated via high fiber levels and 2-dodecyl-6-methoxycyclohexa-2,5-diene-1,4-dione), hypotensive (mediated via apigenin), hypocholesterolemic (mediated via micronized fiber), anti-inflammatory, anti-infective, antitumor, and immune-boosting effects [3].

An important aspect is that the fruit’s ripeness stage affects the composition and content of its antioxidant compounds. It has been reported that week nine is the suggested age for human consumption of Carambola fruit, as consumers can obtain optimal health benefits at this stage [4]. The high antioxidant content of Carambola fruit makes it a potential source of nutritional and pharmaceutical benefits for human consumption. One unexplored alternative is to extract and microencapsulate the fruit’s antioxidant compounds, which provide a protective effect during storage, and additionally reduce their degradation in the digestive tract when the microcapsules are added to the food matrix. It is hypothesized that starchy wall materials produce different protective effects on the antioxidant compounds of Carambola pulp. Therefore, the aim of this study is to evaluate the effect of starchy wall materials on the microencapsulation of Carambola fruit pulp and the retention of the antioxidant characteristics, and the effect of non-extractable polyphenols on the antioxidant properties.

## 2. Materials and Methods

### 2.1. Materials

**Figure 1:**
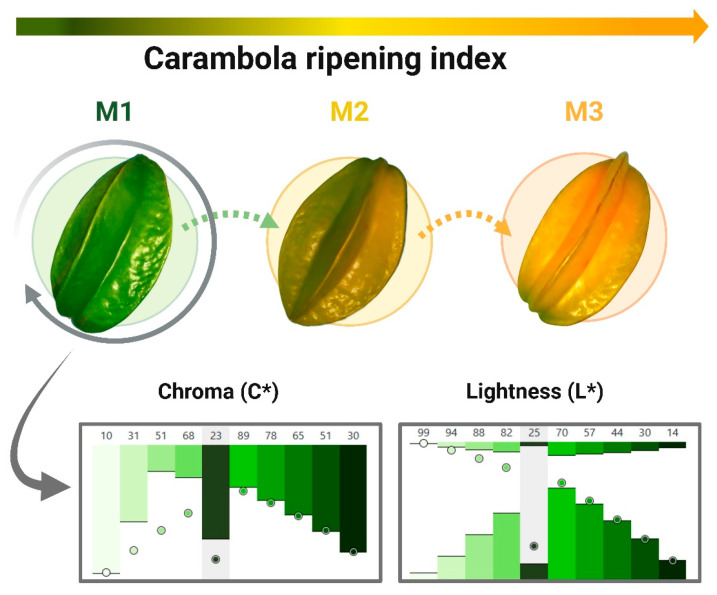
Fruit ripening stages, where M1 = physiological, M2 = organoleptic and M3 = consumption ripeness. The graphs represent the chroma (C*) = 22.55 ± 3.44 and lightness (L*) = 24.78 ± 4.89 content for the selected ripening stage according to the CIE L*a*b color space using CIELab.io open-source library. The data represented corresponds to the mean ± SD of 26 replicates.

Carambola fruits were selected at 11 weeks of growth in the physiological ripening stage (M1 Figure 1) and obtained from “Vivero Yautepec”, Apizaco 33, Felipe Neri, 62732 Yautepec de Zaragoza, Morelos, Mexico. DE 10 (CMD10) and 20 (CMD20) Maltodextrins, as well as commercial starches modified with OSA, namely, N-Lok (CN-LOKS) (Code: 31111101) and Capsul (CCS) (Code: 06560101), were obtained from Ingredion Inc.^®^ (Bridgewater, NJ, USA).

### 2.2. Physicochemical Characteristics of Carambola Fruit

The fruits analyzed were collected on the day of analysis. The polar diameter was measured by taking the maximum length (cm) from the basal to the distal part of the fruit, and the equatorial diameter corresponded to the perpendicular length of the widest part. The color was determined following the International Commission on Illumination Scale using the CIE L*a*b color space with a CR-10 Tristimulus colorimeter, Konica-Minolta, Jenck (Chiyoda, Tokyo, Japan), which measured transmittance and reflectance with a D65 light source. For lightness (L*), L = 0 is black and L = 100 is white. The color saturation, known as chroma (C*), was calculated using cartesian coordinates (a* = Red/Green and b* = Yellow/Blue) according to the following expression:

C* = √ (a2 + b2)(1)

The pulp was extracted by mechanical extraction (crushing) in a Hamilton Beach juicer (67608z Big Mouth Pro, NACCO, Cleveland, OH, USA). Titratable acidity (TA) and soluble solids were measured following the method established by the AOAC [5]. The titratable acidity content was expressed as a percentage of oxalic acid (%) (major organic acid in Carambola pulp; factor: 0.045 g/meq 100 mL pulp). The moisture content was determined following the AACC method [6].

### 2.3. Bioactive Compounds of Carambola Pulp

Polyphenolic compounds were extracted from the pulp using the method proposed by Perez-Jimenez et al. [7] with slight modifications. First, 500 µL of juice was taken and added to 25 mL of methanol–water (50:50) and acidified with 0.8 M *v*/*v* HCl for aqueous–organic extraction. The mixture was then stirred moderately at room temperature (25 °C) for 1 h, and the process was later repeated with a 70% acetone–water solution to obtain low-molecular-weight, extractable polyphenols. The total phenolic content (TPC) was quantified in the aqueous extraction obtained as described by Ruiz-Canizales et al. [8] using the Folin–Ciocalteu method. The absorbance was read at 765 nm. The results were expressed as mg Gallic Acid Equivalent/g db. For flavonoid quantification, the method described by Yan et al. [9] was followed using Aluminum Chloride. The absorbance was read at 430 nm, and the results were expressed as mg Quercetin Equivalent/g db. The residues from the aqueous–organic extraction (non-extractable polyphenols) were hydrolyzed with 10 mL of butanol–HCI with iron (ferric chloride) solution and incubated for 3 h at 100 °C to quantify condensed tannins following the method described by Reed et al. [10]. The absorbance was read at 460 nm. A calibration curve was prepared using Catechin as a standard, and the results were expressed as mg Catechin Equivalent/g db.

### 2.4. Antioxidant Capacity

Free radical scavenging activity was assessed by ABTS, DPPH, and FRAP assays in extractable and non-extractable polyphenols. The activity of 2,2′-azinobis-3-ethylbenzothiazoline-6-sulfonic acid (ABTS) was determined following the methodology proposed by Re et al. [11] and Buratti et al. [12]: First, 20 µL of extract or hydrolysate was mixed with 280 µL of ABTS solution (Abs = 0.7), agitated for 60 s, and incubated at room temperature for 6 min. Finally, intensity was read at 734 nm. The antiradical activity of 2,2-diphenyl-1-picrylhydrazyl (DPPH) was determined according to the procedure described by Bobo-García et al. [13]. First, 20 µL of extract or hydrolysate was mixed with 180 µL of DPPH solution, agitated for 60 s, and incubated in the dark at room temperature for 40 min. The reaction was read at 515 nm. The Ferric Reducing Antioxidant Power (FRAP) assay was performed as described by Benzie and Strain [14] with minor modifications, as described by Ruiz Canizales et al. [8]. Briefly, to prepare the FRAP solution, acetate buffer (300 mM, pH 3.6), TPTZ solution (10 mM in 40 mM of HCl), and ferric chloride hexahydrate (20 mM) were mixed in a 10:1:1 ratio. The activity was measured by reading the absorbance at 20 µL of the extract or hydrolysate, then mixing it with 280 µL of FRAP solution. The mixture was agitated for 60 s and incubated in the dark at room temperature for 30 min. The absorbance was read at 593 nm, where the reduction of ferric iron (Fe^+3^) to ferrous iron (Fe^+2^) was observed. The results were expressed as µmol Trolox Equivalent/g db.

### 2.5. Microcapsule Preparation

Carambola pulp dispersions were prepared with 10% Maltodextrin (DE 10 and 20) and 20% starch (N-Lok and Capsul) and placed in amber flasks to prevent degradation of polyphenolic compounds. Subsequently, the dispersions were placed on a magnetic stirring hot plate (PC-610D, 120V, Corning) at minimum speed. The solids of the Carambola pulp were 8.12%, and the solid concentration in the feed was 18.12% for Maltodextrin and 28.12% for starch. Subsequently, they were individually transferred through a hose to a Buchi B-290 basic mini spray dryer (200 V) under the following conditions: particle size of 1–25 μm, yield of 70%, feed rate of 10%, air pressure of 40–50%, aspiration of 100%, and inlet temperature of 118 °C. The microcapsules obtained were stored in amber glass flasks coated with aluminum and sealed with parafilm tape.

### 2.6. Microcapsule Properties

#### 2.6.1. Encapsulation Efficiency

The total and surface phenolic contents of the microcapsules were determined using the method proposed by Saikia et al. [15], with modifications. The total phenolic content was determined following the method detailed in Section 2.3. To determine the surface phenolic content, 500 mg of a sample was weighted, and 25 mL of methanol:water (50:50) acidified with 0.8 M *v*/*v* HCl was added as the extraction solvent. Samples were stirred for 6 s in a Thermo Scientific Digital Vortex Mixer (Thermo Scientific, Waltham, MA, USA), and the extractable phenolic content was quantified using the Folin–Ciocalteu method (Section 2.3). The encapsulation efficiency (EE) of each microcapsule was determined using the following equation:

EE (%) = (CPC-SPC)/TPC × 100(2)

where CPC is the phenolic content within the capsule core, SPC is the phenolic content on the capsule surface, and TPC is the total polyphenol content in the encapsulates.

#### 2.6.2. Water Activity and Moisture Content

The water activity (aw) was calculated to determine the free water value of the microcapsules, which was measured using AquaLab CX-2 equipment (Decagon Devices Inc., Pullman, WA, USA). The moisture content was determined using the AACC method [6]. The measurements were taken after the pulp was dried and once the powders were already stored in the bakers.

#### 2.6.3. Microcapsule Surface Morphology

The surface morphology was analyzed using scanning electron microscopy (SEM) (EVO LS10, Zeiss, Oberkochen, Germany). The procedure consisted of placing the samples on aluminum supports and carbon-reducing tape, and imaging them at an accelerating voltage of 14 kV and distance of 10 mm using secondary electron microscopy.

#### 2.6.4. Water Sorption in Storage

Humidity adsorption isotherms were performed to determine microcapsule stability using an automated Dynamic Vapor Sorption (DVS) instrument (model DVS-1, Quantachrome Instruments, Boynton Beach, FL, USA) following the method described by Hoyos-Leyva et al. [16]. A 25 mg sample was taken at 25 °C and a relative humidity of 0–0.9%. The instrument was operated using Aquawin 1.11 Software. The adsorption isotherms were created using the Guggenheim–Anderson–De Boer (GAB) model described by the following equation:

X = (Xm C Kaw)/(1 + Kaw) (1 − Kaw + C Kaw)(3)

where X is the moisture content (g of water/100 g of db), aw is the water activity, Xm is the moisture content of the monolayer (g of water/100 g of db), and C and K are constants obtained with the entropic accommodation factor equations.

#### 2.6.5. Particle Size Distribution

The microcapsule particle size distribution was determined using laser diffraction (Mastersizer 2000, Malvern Instruments, Malvern, UK). The sensitivity range was 0.02–2000 μm, and sample preparation was performed as described by Hoyos-Leyva et al. [17].

#### 2.6.6. Thermal Stability

A thermal analysis was performed using a simultaneous thermal analyzer (TGA-DSC, TA Instruments, New Castle, DE, USA), which provides more comprehensive information on material stability and thermal resistance, as well as phase transitions as a function of temperature. The conditions for the sample runs were as follows: a temperature from 25 to 700 °C, a heating rate of 2 °C min^−1^, and 70% excess water.

### 2.7. Stability Study of Polyphenols During Storage

#### Kinetic Reaction Analysis: First-Order Reaction and Half-Life

To assess the stability of the microencapsulated polyphenols and their antioxidant activity, the powders were stored in amber glass flasks at 4, 25, and 35 °C for 63 days [18]. Extractable (TPC and flavonoids) and non-extractable polyphenols (condensed tannins) and their antioxidant capacity (ABTS, DPPH and FRAP) were measured. The results were adapted to first-order kinetics with the equation described below.

Ln (C_0_/Ct) = K_r_ t (4)

t_1/2_ = ln2/K_r_ (5)

where K_r_ represents the rate constant (week^−1^), C_0_ the initial content, and C_t_ the concentration variation.

### 2.8. Statistical Analysis

The statistical analysis of the data followed a randomized experimental design with four treatments. The analyses to determine the fruit’s characteristics were carried out in 26 replicates. For the microcapsules, all analyses were performed in triplicate per treatment to ensure statistical reliability and reproducibility. All results were reported as mean ± standard deviation (SD). To determine statistically significant differences between the results (*p* < 0.05, 95% confidence interval), a one-way analysis of variance (ANOVA) was performed using SigmaPlot software (v.12.0).

## 3. Results

### 3.1. Fruit Characteristics

The maximum length of the harvested fruit was 9.72 ± 0.98 cm, while the equatorial radius range was 6.69 ± 0.53 cm (Table 1 and Figure S2). The weight varied considerably, ranging from 84.61 ± 19.57 g (mass of individual fruits), while the juice content was 89.66 ± 0.57 mL/g fruit. The color of the unripe fruit was predominantly green, shifting to yellow and orange as ripening progressed (Figure 1). The chroma and lightness of the unripe fruit were 22.55 ± 3.44 and 24.78 ± 4.89, respectively. Titratable acidity, measured by oxalic acid content, total and soluble solids, and moisture content, was within the range of values reported in the literature [19]. The pH value was around 2.71, reflecting some fruit with predominantly acidic characteristics due to the accumulation of organic acids during the initial stages of development [20].

### 3.2. Antioxidant Capacity

Carambola pulp shows significant antioxidant capacity, with a total phenolic content of 56.26 mg GAE/g dry weight (Table 2). This result is consistent with that reported by Annegowda et al. [21], who obtained values of 57–60 mg GAE/g dry weight using a water-based extraction method. However, extraction methods using ethanol and methanol as solvents can yield higher yields, up to 97.16 and 142.0 mg GAE/g dry weight, respectively, and so influence the free radical scavenging activities of ABTS, DPPH, and FRAP [22,23].

Vargas-Madriz et al. [24] provided a detailed report on the antioxidant activity of Carambola fruit extracts. The antioxidant capacity, measured by DPPH, was approximately 109.0 μmol TE/g db, which is within the range reported by the authors for the water-based extraction method. Table 2 also shows that extractable polyphenol content was approximately twice that of non-extractable polyphenols, a difference that could be considered during digestion [8].

### 3.3. Morphology and Particle Size

**Figure 2:**
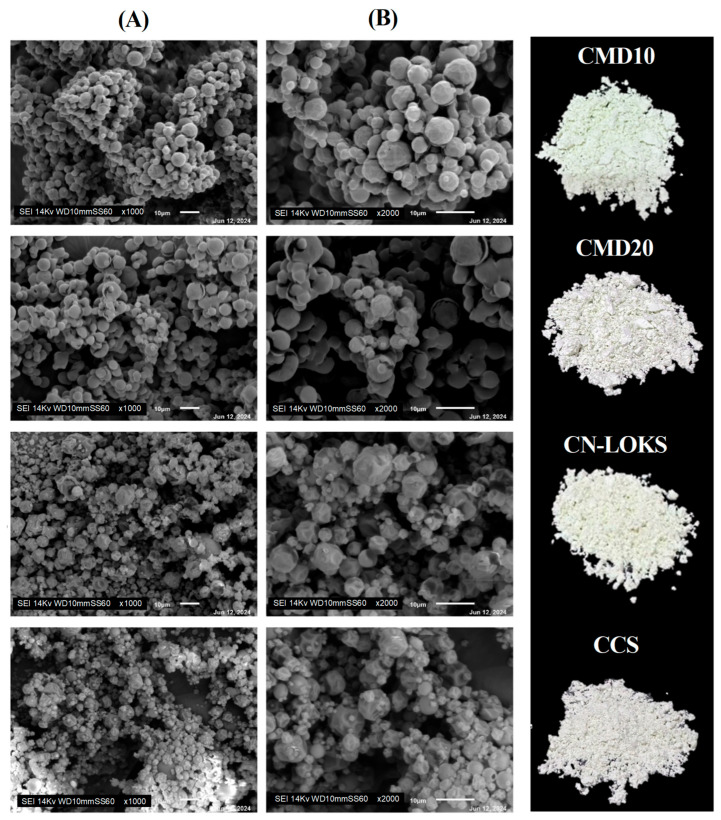
Scanning electron microscopy (SEM) of the superficial morphology of the microcapsules formed at a magnification of (**A**) ×1000 and (**B**) ×2000.

Figure 2 illustrates the morphology of the microcapsules for the four materials used for protection. The microcapsules show a spherical geometry, although with irregularities reflected as flat regions (facets) and possibly small fractures due to the ability of each material to form a surface during the drying process [25]. The CMD10 case is more homogeneous than the other three. The particle size distribution of the wall materials is presented in Figure S3a. N-Lok shows the most bimodal distribution with modes at 0.39 and 3.05 μm. For microcapsules (Figure S3b), the mean particle size is 0.57 μm (CMD10), 0.91 μm (CN-LOKS), 0.39 μm (CMD20), and 0.35 μm (CCS). The incorporation of polyphenols extracted from Carambola significantly reduces the particle size distribution. This pattern is important during the addition of encapsulated powders in a food matrix. For all four cases, the distribution is bimodal, with a mode at 0.3–0.9 μm and a second mode at approximately 1.75–2.25 μm.

### 3.4. Microcapsule Characteristics

The polyphenols extracted from Carambola pulp require protection from adverse light, temperature, and oxygen conditions. Encapsulation efficiency was highest for CN-LOKS (81.4%) and lowest for CCS (59.6%) (Table 3). However, there was a negative correlation between encapsulation efficiency and TPC retention, showing no increase in TPC with rising EE (ρ = −0.6069; significance = 0.3130). The wall material influenced the EE, with the polyphenol content at the surface determining this value. One starch (N-LOKS) and one Maltodextrin (CM10) wall material showed the highest EE values; the structure of the starchy wall material was related to this pattern and could be selected to protect the bioactive compounds in Carambola pulp. The ratio of TPC retained in the bulk to the surface was approximately 4:1. The moisture content was no greater than 4.1%, with CN-LOKS exhibiting the lowest value (3.27%). Water activity was similar for all four microcapsules, with a maximum value of 0.42, reflecting the stability of water molecules within the wall material structure [26].

### 3.5. Adsorption Isotherms

The microcapsule adsorption isotherms are shown in Figure S4. The isotherms show monotonic growth with water activity, indicating a Type III isotherm. This type of isotherm describes a process in which the adsorbate has a similar affinity for the adsorbent and for itself, resulting in a convex curve towards the pressure axis that favors the formation of multilayers without a defined monolayer, indicating strong lateral interactions between adsorbed molecules and weak interactions with the surface. The GAB model was used to fit the adsorption isotherms via least squares. The estimated parameters are presented in Table 4. The CN-LOKS and CMD10 microcapsules showed the minimum (18.897 g H_2_O 100g^−1^ db) and maximum (24.961 g H_2_O 100g^−1^ db) monolayer capacities. However, the energy constant was lower (0.437) for CMD20 and higher (0.517) for CN-LOKS. This means that, except for the CMD20 microcapsule, the materials presented similar energy barriers to the adsorption process.

### 3.6. Thermal Analysis

Figure S5 presents the results of the microcapsule thermal analysis (TGA and DSC). The Maltodextrin TGA curves showed a small decrease in weight around 100 °C, corresponding to the loss of free water, and a loss of wall material mass between 252 and 276 °C. The short-chain maltodextrin (CMD20) showed a higher degradation temperature due to its more compact microcapsule structure [27]. The wall materials based on starch (CN-LOKS and CCS) showed differences in the TG pattern; CN-LOKS showed the lowest degradation temperature, and CCS a degradation temperature lower than CMD20. The structure of the wall material influenced the compactness of the microcapsule. The DSC analysis of CMD10 and CCS microcapsules showed two phase transitions: the first around 100 °C and the second at 296 °C and 340 °C, respectively. The first one was attributed to polyphenol degradation, and the second one to degradation of the biopolymer matrix. An increase in the trend of the heat flow curve was seen at around 300 °C, indicating an accelerated breakdown of glucose chain bonds. This point is consistent with the TGA analysis, which showed a rapid decrease in weight around this temperature. In summary, the four microcapsules exhibit similar thermal properties.

### 3.7. Stability of Polyphenols in Microcapsules

**Figure 3:**
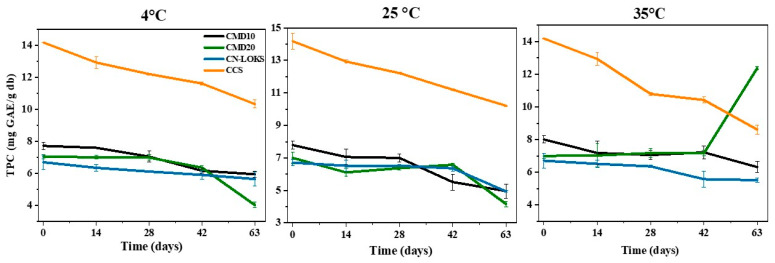
Degradation kinetics of total phenolic content (extractable polyphenols) in microcapsules stored at 4, 25, and 35 °C. The values correspond to the means of three replicates. Carambola pulp with CMD10: Maltodextrin 10; CMD20: Maltodextrin 20; CN-LOKS: N-Lok; and CCS: Capsul starch. db: dry basis.

**Figure 4:**
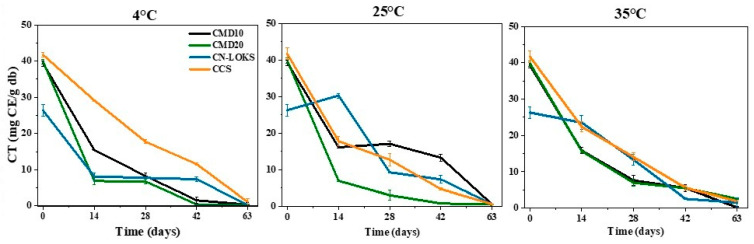
Degradation kinetics of condensed tannins (non-extractable polyphenols) in microcapsules stored at 4, 25, and 35 °C. The values correspond to the means of three replicates. Carambola pulp with CMD10: Maltodextrin 10; CMD20: Maltodextrin 20; CN-LOKS: N-Lok; and CCS: Capsul starch. db: dry basis.

Figure 3 details the temporal stability results of extractable polyphenols at different storage temperatures (4, 25, and 35 °C). Antioxidant stability was evaluated using ABTS+, DPPH, and FRAP assays. In general, polyphenol levels decreased with temperature, indicating that they were exposed to more adverse conditions when microcapsules were stored for prolonged periods at different temperatures. CN-LOKS microcapsules showed the highest half-life (38.50 days), and CMD20 microcapsules had the lowest (21.66 days) for TPC at a storage temperature of 4 °C. However, CMD10 microcapsules provided the best protection for flavonoids, with a half-life of 25.67 days. The best protection of antioxidant activity, as measured by ABTS+, was obtained with CCS (63.01 days). In contrast, CMD10 microcapsules showed the longest mean half-life (17.77 days) for DPPH. However, CCS showed the longest half-life (40.77 days) for antioxidant activity quantified by the FRAP assay. These results show that no microcapsule was effective at protecting active compounds across all their properties. Comparable results were obtained for the other storage temperatures and for the non-extractable polyphenols and their antioxidant capacity (Figure 4).

### 3.8. Correlation Analysis

**Figure 5:**
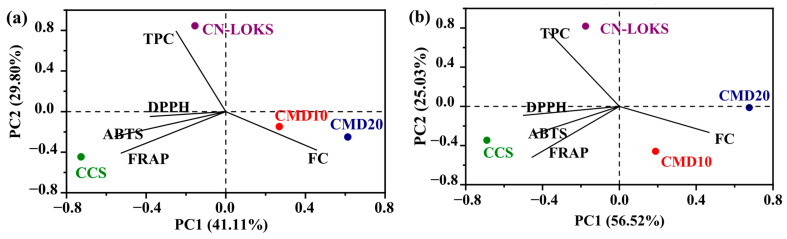
PCA of microencapsulated extractable polyphenols stored at (**a**) 4 and (**b**) 25 °C. Carambola pulp with CMD10: Maltodextrin 10; CMD20: Maltodextrin 20; CN-LOKS: N-Lok; and CCS: Capsul starch.

Antioxidant activity involves five different indices, including TPC, FC, ABTS, DPPH and FRAP. These indices are monitored to assess the stability of encapsulated extracts. However, Figure 4 suggests that the variations in the antioxidant activity indices are not fully independent. Table 5 displays the pairwise Pearson’s correlation coefficient of microencapsulated extractable polyphenols stored at 4 °C. Strong correlations, with an absolute value higher than 0.5, are depicted in boldface. From ten pairwise correlations, six exhibit strong correlations, suggesting codependence between the indices. Interestingly, TPC shows strong correlations only with FC, whilst it has weak correlations with the other three antioxidant activity indices. In turn, one concludes that tracking the evolution of TPC does not suffice to assess the antioxidant stability of the microcapsules. Similar results were found for microcapsules stored at 25 °C (Table 6), although stronger correlations were detected. In particular, FRAP seems to be a good index as it is strongly correlated with the other four indices. A complementary picture of correlations can be found with a multivariate analysis. Indeed, principal component analysis (PCA) can corroborate the pairwise analysis and at the same time provide further insights into the multiple codependences of antioxidant capacity indices. The motivation is to detect multivariate correlations that provide practical insights for the characterization and monitoring of antioxidant activity in extracts and encapsulates. To this end, the half-life of the four microcapsule formulations was considered. Figure 5a shows the results for the two main extractable components. These two main components contribute 70.91% of the half-life variability. Interestingly, TPC and FC are anti-correlated, meaning that the half-life of TPC is inversely proportional to the half-life of FC. On the other hand, the half-life of antioxidant activity (ABTS, DPPH, and FRAP) forms a group that is perpendicular to TPC and FC. This means that antioxidant activity stability is not correlated with total phenol and flavonoid content stability.

**Figure 6:**
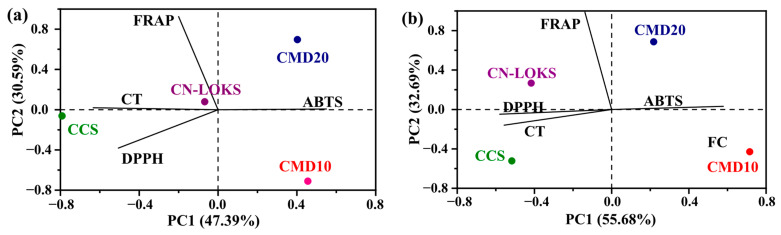
PCA non-extractable polyphenols and antioxidant capacity of microencapsulated Carambola pulp stored at (**a**) 4 and (**b**) 25 °C. Carambola pulp with CMD10: Maltodextrin 10; CMD20: Maltodextrin 20; CN-LOKS: N-Lok; and CCS: Capsul starch.

A consequence of this lack of correlation is that the phenol content in the extract does not guarantee acceptable antioxidant activity. Also, monitoring the extraction of phenolic compounds is not sufficient, requiring more specific analysis of active compounds with antioxidant activity. The CMD10 and CMD20 microcapsule formulations promote FC stability, while the CN-LOKS formulation promotes TPC stability. The CCS formulation promotes the temporal stability of antioxidant activity. This trend also holds for a storage temperature of 25 °C (Figure 5b). The PCA results for non-extractable polyphenols and antioxidant activity are presented in Figure 6a,b for 4 and 25 °C, respectively. This scenario differs from the earlier case. CT, ABTS, and, to a lesser extent, DPPH show alignment. However, ABTS is anti-correlated with CT and DPPH. FRAP does not show alignment with the other three indicators. In terms of formulations, CMD10 and CMD20 contribute to ABTS stability, although the alignment is not extraordinarily strong. The CN-LOKS formulation is found at the origin, suggesting that it does not significantly affect the stability of the indicators. Summing up, the correlation analysis shows that the antioxidant stability of microcapsules can be assessed by monitoring only one or two indices, with FRAP as a potentially practical index. For instance, FRAP and TPC could be considered for monitoring, as the other two antioxidant capacity indices are strongly correlated with FRAP. It should be clarified that it is not mathematical independence being discussed but practical interdependence. The presence of clusters indicates that the variables contained within them can be monitored using a single variable, either physical or a combination of variables. Such clustering facilitates the monitoring and evaluation of encapsulation, since it only requires focus on a reduced number of variables. This point was commented on in the revised manuscript.

## 4. Conclusions

This study characterized microcapsules of Carambola pulp using four starchy wall materials. The EE varied with the wall material, and the particle size of the microencapsulated material was smaller than that of the spray-drying wall material. The analysis of thermal and isotherm results showed the stability of the microcapsules. The antioxidant activity of the polyphenols in the microcapsules was retained, showing the feasibility of producing a functional ingredient. The choice of wall material depends on the specific polyphenol and antioxidant activity factors that are to be promoted. The results obtained for the antioxidant characteristics and storage stability of encapsulated Carambola pulp suggest applications in yogurt, smoothies, salads, and fruits, where the food matrix is not important, and the encapsulated material can be used in food matrices produced during processing, such as pasta, bakery products, and snacks.

The main limitations of the present study are the following: (a) studies on the digestibility of capsules in terms of the release of biocomponents are required to have a vision of the encapsulation efficiency of the encapsulation systems; (b) more detailed characterization of the encapsulation systems should provide information on the interactions, physical or chemical, of the biocompounds with the encapsulation material; (c) and finally, it is necessary to evaluate the performance of the encapsulations in a food matrix, such as yogurt, cheese, or bread.

## Figures and Tables

**Table 1:** Physicochemical properties of Carambola fruit at physiological ripening.

Characteristics	M1
Polar diameter (cm)	9.72 ± 0.98
Equatorial diameter (cm)	5.69 ± 0.53
Weight (g)	84.61 ± 19.57
Juice volume (mL/100 g fruit)	89.66 ± 0.57
Chroma (C*)	22.55 ± 3.44
Lightness (L*)	24.78 ± 4.89
Titratable acidity (% oxalic acid)	0.34 ± 0.01
Total solids (%)	8.12 ± 0.38
Soluble solids (°Brix)	6.80 ± 0.10
Moisture (%)	91.87 ± 0.39
Hydrogen potential (pH)	2.71 ± 0.10

M1 = physiological ripeness of Carambola fruit. Data corresponds to the mean ± SD of 26 replicates. Total solids content (%) was obtained by comparing the moisture content with the total solids content. Oxalic acid factor: 0.045 g/meq/100 mL of juice. The C* and L* values were obtained according to the CIE L*a*b color space using the CIELab.io open-source library.

**Table 2:** Extractable and non-extractable polyphenols and antioxidant capacity of Carambola pulp.

Analysis	Content
Extractable polyphenols (EPs)	
Total phenolic content (mg GAE/g db)	56.26 ± 1.37
Flavonoid content (mg QE/g db)	5.04 ± 0.44
Antioxidant capacity (µmol TE/g, db)	
ABTS+	65.61 ± 0.10
DPPH	109.37 ± 0.60
FRAP	83.90 ± 0.07
Non-extractable polyphenols (NEPs)	
Condensed tannins (mg CE/g db)	24.85 ± 2.37
Antioxidant capacity (µmol TE/g, db)	
ABTS+	31.84 ± 1.52
DPPH	36.40 ± 1.24
FRAP	50.47 ± 0.16

Data corresponds to the mean ± SD of three replicates. Dry basis. GAE: Gallic Acid Equivalent; QE: Quercetin Equivalent; TE: Trolox Equivalent; CE: Catechin Equivalent; ABTS: 2,2-azino-bis (3-ethylbenzothiazoline-6-sulfonic acid); DPPH: 2,2-diphenyl-1-picrylhydrazyl; and FRAP: Ferric Reducing Antioxidant Power.

**Table 3:** Physical and functional properties of Carambola pulp microcapsule powders with different wall materials.

Microcapsule	TPC Retention		EE (%)	Moisture (%)	Aw
	Total	Surface			
CMD10	7.06 ± 0.23 ^d^	0.97 ± 0.04 ^c^	75.8 ± 1.2 ^ab^	4.1 ± 0.54 ^a^	0.41 ± 0.00 ^b^
CMD20	7.00 ± 0.14 ^cd^	1.44 ± 0.09 ^d^	65.9 ± 0.8 ^a^	4.41 ± 0.50 ^a^	0.42 ± 0.00 ^a^
CN-LOKS	6.36 ± 0.41 ^bc^	0.65 ± 0.04 ^b^	81.4 ± 0.2 ^b^	3.27 ± 0.24 ^b^	0.42 ± 0.01 ^a^
CCS	14.18 ± 0.01 ^a^	3.59 ± 0.07 ^a^	59.6 ± 0.5 ^a^	4.5 ± 0.05 ^a^	0.42 ± 0.00 ^a^

TPC: total phenolic content (mg GAE/g db); EE: encapsulation efficiency; Aw: water activity. Data corresponds to the mean ± SD of three replicates, except for Aw (ten replicates). Different small letters in each column denote ANOVA statistical differences (*p* < 0.05). Dry basis. Carambola pulp with CMD10: Maltodextrin 10; CMD20: Maltodextrin 20; CN-LOKS: N-Lok; and CCS: Capsul starch.

**Table 4:** GAB model parameters for microcapsules with different wall materials.

Microcapsules	X_m_ (g H_2_O 100g^−1^ db)	C	K
CMD10	24.961 ± 1.118 ^a^	0.515 ± 0.018 ^a^	0.543 ± 0.019 ^b^
CMD20	22.356 ± 1.084 ^ab^	0.437 ± 0.019 ^b^	0.601 ± 0.021 ^a^
CN-LOKS	18.897 ± 1.016 ^c^	0.509 ± 0.017 ^a^	0.587 ± 0.018 ^ab^
CCS	20.273 ± 1.235 ^b^	0.517 ± 0.023 ^a^	0.545 ± 0.016 ^b^

Means in each column followed by different letters are significantly different (*p* < 0.05). Carambola pulp with CMD10: Maltodextrin 10; CMD20: Maltodextrin 20; CN-LOKS: N-Lok starch; and CCS: Capsul starch. C, Xm, and K denote the energy constant, the monolayer moisture capacity, and the correction factor; db: dry basis.

**Table 5:** Pairwise Pearson’s correlations of microencapsulated extractable polyphenols stored at 4 °C. The value in parentheses indicates the two-tailed statistical significance. Bold numbers indicate strong pairwise correlations.

Sample	TPC	FC	ABTS	DPPH	FRAP
**TPC**	1.00 (--)	**0.61 (0.39)**	0.11 (0.88)	0.37 (0.62)	−0.03 (0.96)
**FC**	--	1.00 (--)	**0.70 (0.29)**	−0.11 (0.88)	**−0.51 (0.49)**
**ABTS**	--	--	1.00 (--)	0.47 (0.52)	**0.96 (0.03)**
**DPPH**	--	--	--	1.00 (--)	**0.57 (0.42)**
**FRAP**	--	--	--	--	1.00 (--)

**Table 6:** Pairwise Pearson’s correlations of microencapsulated extractable polyphenols stored at 25 °C. The value in parentheses indicates the two-tailed statistical significance. Bold numbers indicate strong pairwise correlations.

Sample	TPC	FC	ABTS	DPPH	FRAP
**TPC**	1.00 (--)	**0.73 (0.26)**	**0.55 (0.44)**	0.33(0.66)	**0.61 (0.39)**
**FC**	--	1.00 (--)	**−0.57 (0.42)**	**−0.73 (0.27)**	**−0.94 (0.05)**
**ABTS**	--	--	1.00 (--)	**0.84 (0.15)**	**0.76 (0.23)**
**DPPH**	--	--	--	1.00 (--)	**0.91 (0.08)**
**FRAP**	--	--	--	--	1.00 (--)

## Data Availability

The original contributions presented in this study are included in the article/Supplementary Materials. Further inquiries can be directed to the corresponding author.

## Supplementary Materials

The following supporting information can be downloaded at: https://www.mdpi.com/article/10.3390/foods15101699/s1, Figure S1: (A) Three and plantation illustration. (B) Flower buds, anthesis, and preanthesis of the flower. (C) Alternate evergreen branches and leaves. (D) Fruits in clusters, (E) Physiologically ripe fruits. (F) Star-shaped equatorial sections, Figure S2: Polar (left panel) and equatorial (right panel) diameters of typical Carambola fruits. Values correspond to mean of 26 replicates, Figure S3: Particle size distribution of (A) Controls (wall materials) and (B) Carambola pulp microcapsules with CMD10: Maltodextrin 10, CMD20: Maltodextrin 20, CN-LOKS: N-Lok and CCS: Capsul starch. Values correspond to mean of three replicates, Figure S4: Adsorption isotherms at 25 °C of Carambola pulp microcapsules with CMD10: Maltodextrin 10, CMD20: Maltodextrin 20, CN-LOKS: N-Lok and CCS: Capsul starch. Values correspond to mean of three replicates, Figure S5: TGA (continuous line) and DSC (dashed line) curves represent the thermal behavior of the different microcapsules. Values correspond to the mean of three replicates. Carambola pulp with CMD10: Maltodextrin 10, CMD20: Maltodextrin 20, CN-LOKS: N-LOK, and CCS: Capsul starch.
