# Supplementary material for: Effect of Starchy Wall Materials in the Microencapsulation of Carambola Fruit (Averrhoa carambola) Pulp: Antioxidant Characteristics
Source: Foods. 2026 May 12;15(10):1699. doi: 10.3390/foods15101699 (PMC13206637; doi:10.3390/foods15101699)
Supplement: Supplementary file 1 [file foods-15-01699-s001.zip › foods-4243899-supplementary.pdf]

Supplementary Materials

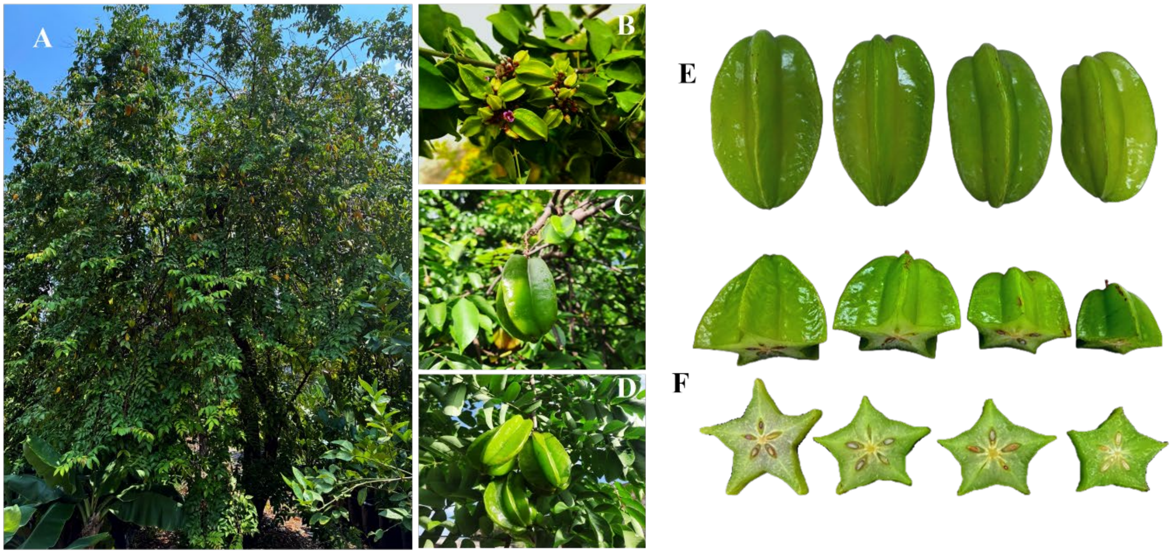

Figure S1. (A) Three and plantation illustration. (B) Flower buds, anthesis, and preanthesis of the flower. (C) Alternate evergreen branches and leaves. (D) Fruits in clusters, (E) Physiologically ripe fruits. (F) Star-shaped equatorial sections.

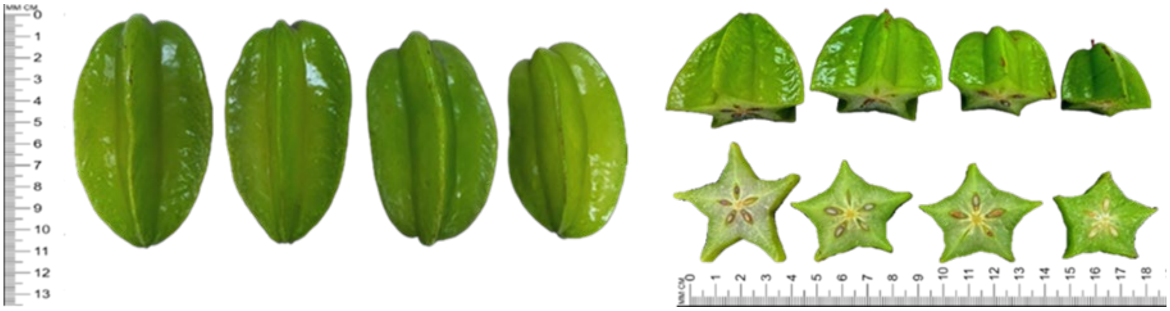

Figure S2. Polar (left panel) and equatorial (right panel) diameters of typical Carambola fruits. Values correspond to mean of 26 replicates.

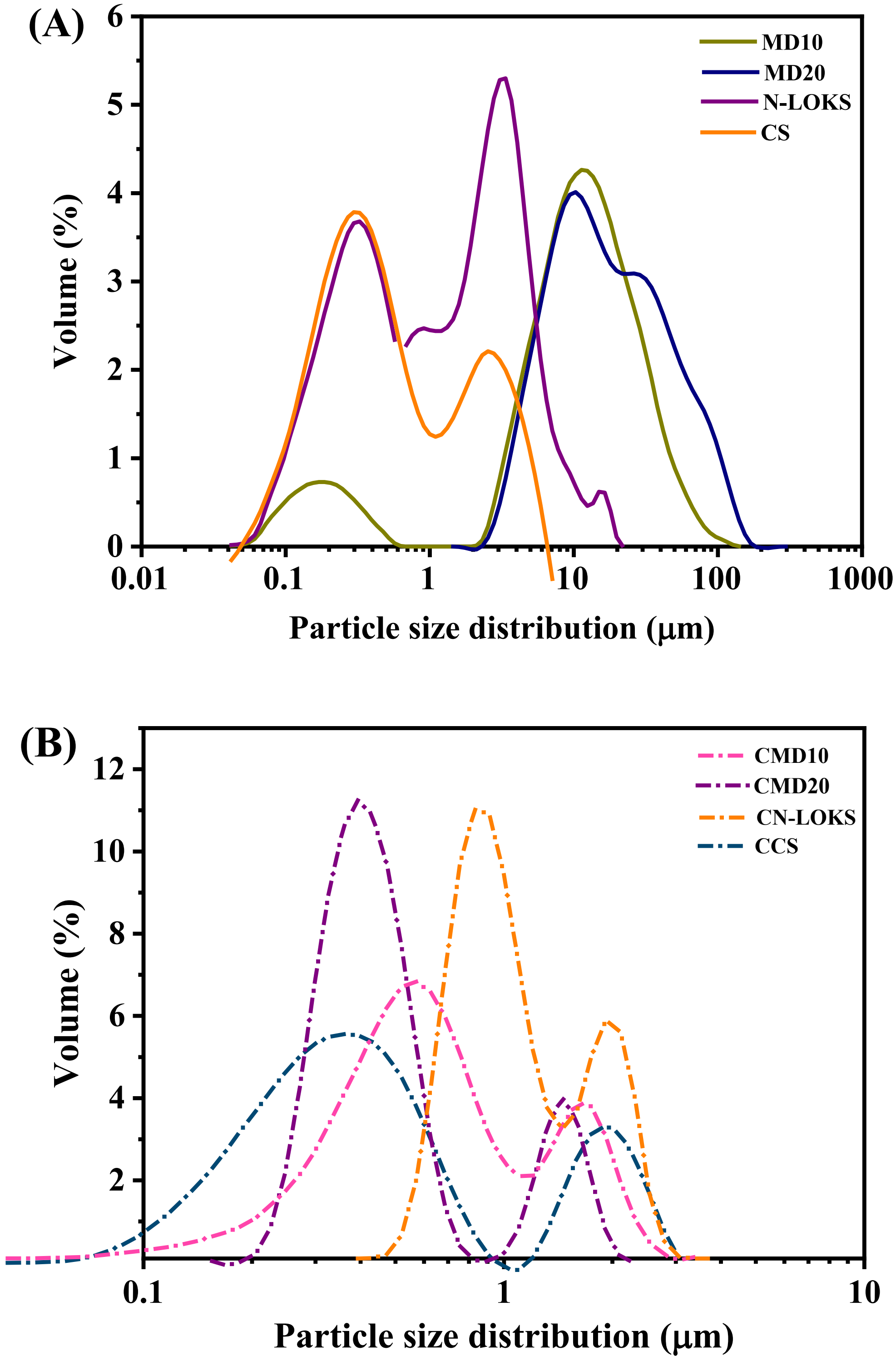

Figure S3. Particle size distribution of (**A**) Controls (wall materials) and (**B**) Carambola pulp microcapsules with CMD10: Maltodextrin 10, CMD20: Maltodextrin 20, CN-LOKS: N-Lok and CCS: Capsul starch. Values correspond to mean of three replicates.

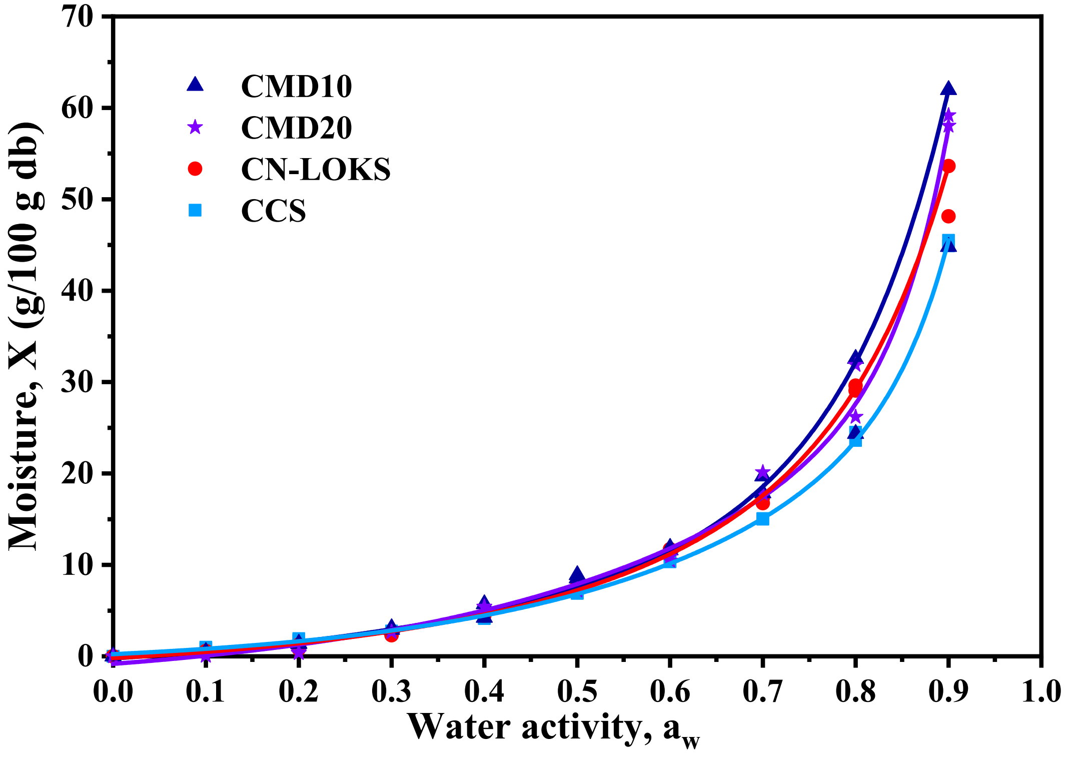

Figure S4. Adsorption isotherms at 25 °C of Carambola pulp microcapsules with CMD10: Maltodextrin 10, CMD20: Maltodextrin 20, CN-LOKS: N-Lok and CCS: Capsul starch. Values correspond to mean of three replicates.

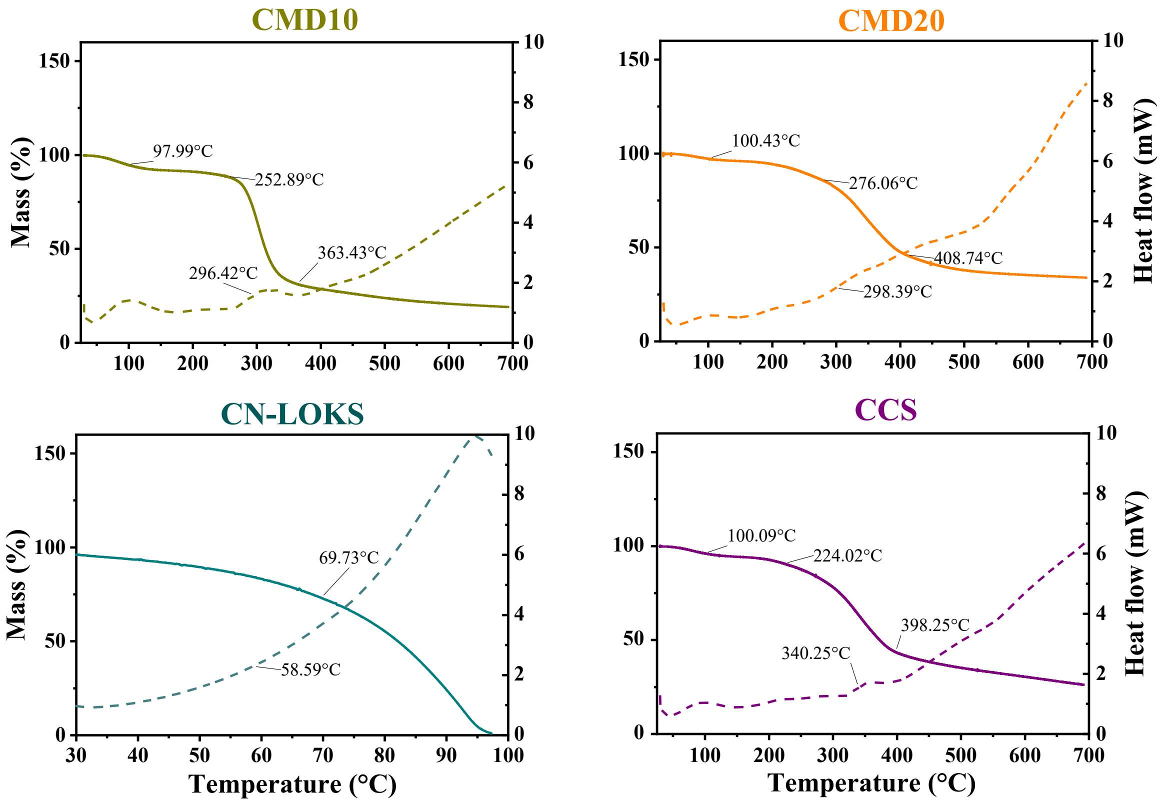

Figure S5. TGA (continuous line) and DSC (dashed line) curves represent the thermal behavior of the different microcapsules. Values correspond to the mean of three replicates. Carambola pulp with CMD10: Maltodextrin 10, CMD20: Maltodextrin 20, CN-LOKS: N-LOK, and CCS: Capsul starch.
